# The Use of Simulation Models and Student-Owned Animals for Teaching Clinical Examination Procedures in Veterinary Medicine

**DOI:** 10.3390/vetsci10030193

**Published:** 2023-03-04

**Authors:** Ricardo Marcos, Sónia Macedo, Macamen de Vega, Pablo Payo-Puente

**Affiliations:** 1 Cytology and Hematology Diagnostic Services, Laboratory of Histology and Embryology, Department of Microscopy, ICBAS—School of Medicine and Biomedical Sciences, University of Porto (U.Porto), Rua de Jorge Viterbo Ferreira, 228, 4050-313 Porto, Portugal; 2 Centro de Investigação Vasco da Gama (CIVG), Escola Universitária Vasco da Gama (EUVG), Campus Universitário, Av. José R. Sousa Fernandes, 3020-210 Coimbra, Portugal; 3 GIIPEV-Grupo Investigação em Ensino de Medicina Veterinária, Rua de Jorge Viterbo Ferreira 228, 4050-313 Porto, Portugal; 4 Department of Veterinary Clinics, School of Medicine and Biomedical Sciences ICBAS-UP, Rua de Jorge Viterbo Ferreira 228, 4050-313 Porto, Portugal

**Keywords:** educational methodology, simulator, clinical examination

## Abstract

**Simple Summary:**

Clinical examination procedures (CEPs) are cornerstone skills for veterinarians that are taught in all veterinary faculties. CEPs include procedures that are well tolerated by animals and others that are not. In a classical teaching approach, institutional animals which are kept in kennels at the university are used to teach and practice CEPs. Undergraduate students (n = 231) from four consecutive years were assigned to two groups that used institutional animals only (AO) or a combination of students’ owned animals and simulation models (model–animal, MA) to teach and practice CEPs. The latter comprised stuffed dogs and handmade molding silicone models. The learning outcome of each system was compared through questionnaires, grades, and pass rates in objectively structured clinical examinations. Most veterinary students had their own animals, and it was easy to have a dog per group of two students in class. All the students’ owned animals adapted well to this environment. The interest in the practical activities with the simulation models was comparable to that exhibited in the AO system, and students reported to learn more with the MA method. No differences existed in the final grades and pass rates. The MA system was effective for learning CEPs. Beyond animal welfare advantages, the MA system increased out-of-school training and had financial saving benefits.

**Abstract:**

Clinical examination procedures (CEPs) are cornerstone clinical skills for veterinary practitioners, being taught in all veterinary faculties. CEPs include innocuous procedures that are well tolerated by animals as well as more distressful and less tolerated ones. In a classical approach, institutional animals are used to teach and practice CEPs. Two hundred and thirty-one undergraduate students from four consecutive years were assigned to two groups that used institutional animals only (AO) or a combination of students’ owned animals and simulation models (model–animal, MA) to teach and practice CEPs. This latter comprised stuffed teddy dogs, eye and ear models made of molding silicone, as well as skin models. The learning outcome of each system was compared through questionnaires (throughout classes and at the end of course), grades, and pass rates in objectively structured clinical examinations. Most veterinary students had their own animals, being easy to have a dog per group of two students in class. All the students’ owned animals adapted well to this environment. The interest in the practical activities with the simulation models was comparable to that exhibited in the classical AO system. Students reported to learn more with the MA system than with the AO, while the interest on the subjects and the relevance were appraised similarly in both systems. No differences existed in the final grades and pass rates. The MA system was effective for learning CEPs. Beyond animal welfare advantages, this system increased the out-of-school training and had financial saving benefits, being a valuable option for the teaching and training of CEPs.

## 1. Introduction

Clinical examination procedures (CEPs) are cornerstone clinical skills for veterinary practitioners and serve as a baseline for all internal medicine and clinical activities. These procedures are taught in all veterinary faculties, being mandatory practical competences for all veterinary students [1]. Depending on the veterinary curricula, CEPs may be taught either in the second or third year of the veterinary degree. CEPs are needed to recognize signs of illness in animals. CEPs include innocuous procedures that are fairly well tolerated by animals, such as auscultation or lymph node palpation, as well as distressful and less tolerated procedures, such as otoscopy, fine needle aspiration (FNA), or many ophthalmology tests. 

It is well recognized that a major investment in learning basic skills, such as CEPs, will save many hours of auxiliary teaching in future practical contexts and offers major benefits by developing the practical confidence of students [2]. In fact, the lack of self-confidence by students has been described as the most common source of negative emotions when a practical skill must be performed [3]. Learning CEP (e.g., exploring the clinical signs of an animal within a reasonable time) is similar to any other practical knowledge such as music, sculpture, or even surgery. Besides knowing how things are performed in an abstract way (knowledge), students need to spend their time “learning how to do” (skills learning) and repeatedly practice the procedures, so that the CEP can be performed systematically and quickly (intrinsic skills). To analyze the learning effectiveness of CEP, the Kirkpatrick’s training evaluation framework can be used [4]. This model comprises distinct levels of evaluation, from assessing the students’ reactions and learning in the first and second levels, to addressing changes in the behavior and output results at the third and fourth levels, respectively. The first and second levels are also called internal criteria, being the focus of most studies on training programs, in opposition to the third and fourth levels, the so-called external criteria, that typically occur after the program [5].

For teaching CEPs, two main sets of items are usually needed: exploration tools (e.g., thermometers, otoscopes, ophthalmoscopes, reflex hammers, containment systems, stethoscopes) and animals, which serve as “models” for the skill learning. Currently, there is a trend towards reducing the use of animals in teaching procedures in many veterinary faculties [6,7,8]. It is estimated that up to 10% of the animals listed for experimental purposes are used for education and training [9]. Traditionally, live animals used for education purposes have been derived from three sources: animals seen at the university clinics, shelter animals, and purpose-bred animals [10,11]. Universities frequently buy dogs of specific breeds (e.g., Beagles) [10] from authorized breeders that are used for both animal research and learning activities. This has important ethical connotations because animals will be forced to live all, or almost all, of their existence in a university kennel, which often has limited space.

In recent years, there has been intense research on the use of non-animal models for learning various subjects [6]. Some universities around the globe, from the Ankara University to the University of Hannover or the University of California Davis, for example, already have a long history of the so-called “Clinical Skill Labs”, where plastic models and full-body manikins are used to learn and practice various procedures [8]. Even if it has been shown that the learning outcomes with non-harmful teaching methods can be similar to the use of harmful animal models [12], it is still advocated among veterinary educators that practicing on living animals is needed for proper learning [7]. For learning purposes, model-based and animal-based approaches are often grounded on polarized opinions, according to their advantages and drawbacks [7]. To the best of our knowledge, there are no studies focusing on the use of a mixed model–animal (MA) system, which, at least in theory, could incorporate the best factors of each polarized opinion.

The aim of this study was to evaluate the use of an innovative solution for teaching CEPs to veterinary students and compare it with a traditional “animals only” (AO) solution. The mixed MA system was comprised of simulation models used for harmful and distressful procedures and student-owned animals used for non-stressful activities.

## 2. Materials and Methods

### 2.1. Experimental Design

**Figure 1:**
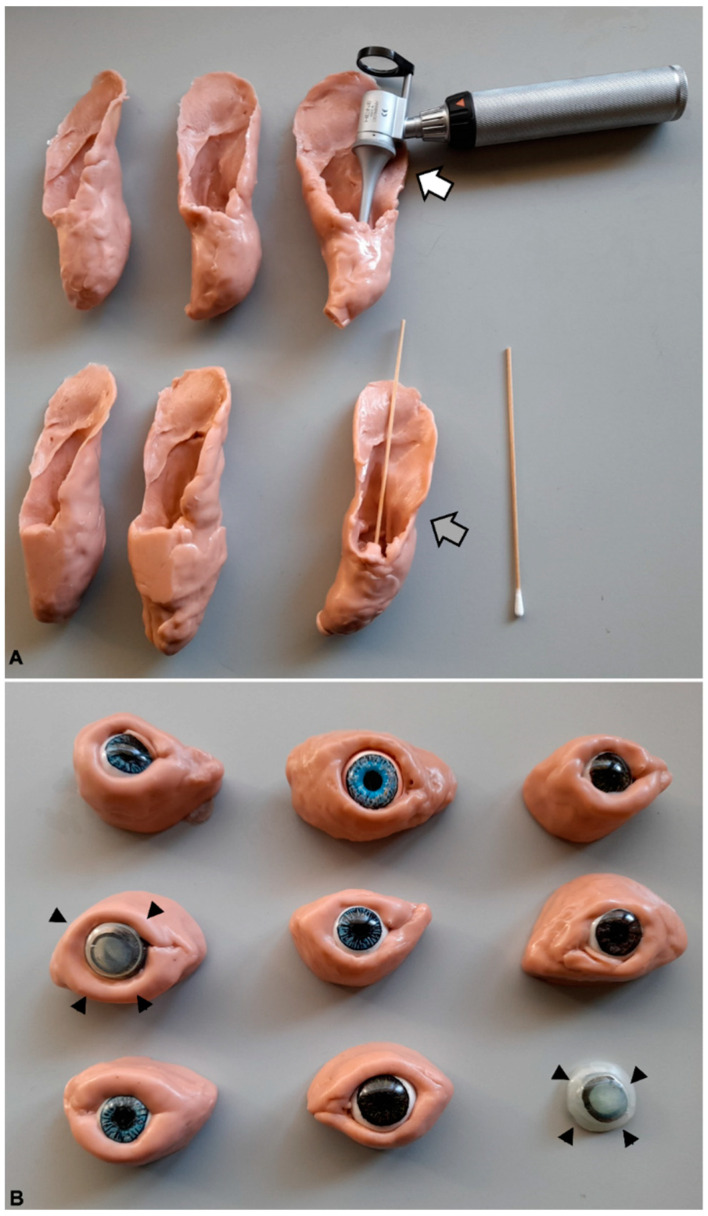
Ear (**A**) and eye (**B**) models made with moldable silicone. The ear models are at scale (medium sized dog) and allowed otoscopy (white arrow) and ear cytology (grey arrow) procedures. The eye models included the eye and eyelids; the corneas could be changed to represent various clinical conditions, such as corneal edema (arrowheads).

**Figure 2:**
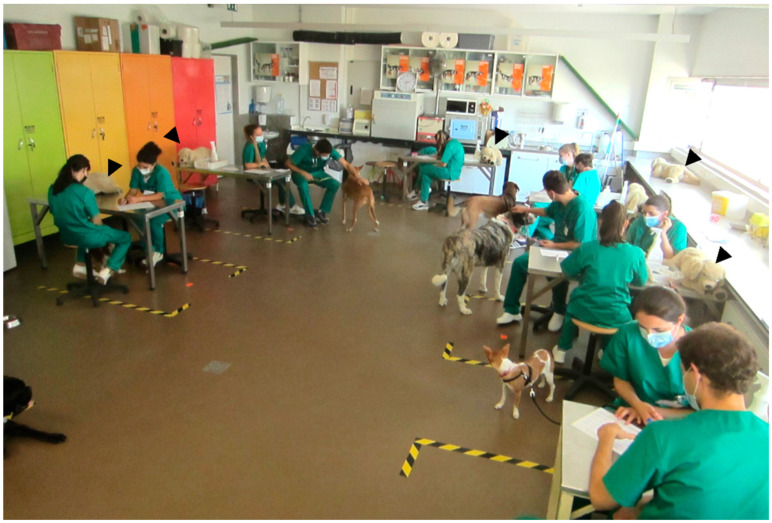
Students in class with their own animals of different sizes and breeds. Each group of two or three students first practiced the procedures with a stuffed teddy dog (arrowheads) and then with their own dog.

To evaluate the performance of the MA system, a case-control monocentric study was performed. Two hundred and thirty-one undergraduate third-year students in a Doctorate in Veterinary Medicine (DVM) program were assigned to two groups in four consecutive years (N1 to N4). In the control group, [N1(2018/19) 57 students and N2(2019/20) 53 students] only institutional animals (Beagles) were used in practical “learning-by-doing” classes. In this AO group, all in-class CEPs were practiced only with institutional animals. The case group [N3(2020/21) 61 students and N4 (2021/22) 60 students] adhered to the MA approach, using low-cost models for distressful procedures (Figure 1), whilst conducting non-stressful procedures using student-owned dogs (Figure 2). These four consecutive years were comparable in terms of gender distribution [17% and 15% males in N1(2018/19) and N2(2019/20), respectively, and 21% and 23% males in N3(2020/21) and N4 (2021/22), respectively] and age of students [21 years old in both groups, range 20 to 34 years old].

To assess the students’ perception of the learning methodology, anonymous questionnaires were obtained. Two types were used: brief questionnaires with a single question, which were distributed after each class, and long questionnaires that were filled after the final examination. Both questionnaires included questions with Likert scales (1 to 5 scale, 3 being the average) which appraised the amount of learning, the interest and relevance of classes, as well as issues regarding the final examination (difficulty, stringency, and fairness). Answers to the questionnaires were always voluntary and anonymous, and personal data were never collected.

In both the case (MA) and control (AO) groups, an objectively structured clinical examination (OSCE) was performed at the end of the semester using the same evaluator (PP), who has more than 20 years of experience with OSCE evaluations. The same type of OSCE evaluations were performed for the two groups (case and controls). The use of institutional animals in classes was always preceded by the authorization of the Institutional Animal Welfare Committee (P328/2019/ORBEA and P347/2020/ORBEA). The collection of data from students was authorized by competent local authorities [Comissão de Ética 2022/CE(413/2023/CETI)].

### 2.2. Simulation Models

The simulation models comprised stuffed teddy dogs (Gosig Golden, IKEA, Almhult, Sweden), which were used in basic procedures (restraints and positioning for different types of exams) and in some painful procedures and tests (e.g., cystocentesis, deep sensitivity tests). For ophthalmology and otoscopy procedures, eye and ear models were built in-house, made of molding silicone (Herbitas Blanda Blanda, Nakamor Gel Corporation, Valencia, Spain) (Figure 1). The ear model allowed the practice of sampling for cytology purposes, as well as for otoscopy, with different scenarios, such as inflammation and ruptured tympanum and the presence of foreign bodies. Regarding the eye models, these included the eyelids and eye, with interchangeable corneas that simulated various clinical conditions, such as corneal edema (Figure 1), cornea stromal hemorrhage with neovascularization, and anterior chamber hemorrhage (hyphema). For dermatology, skin models were used to teach skin biopsies and skin scrapings, as well as models for FNA [13]. The latter comprised two types of models, namely, boxes covered with artificial fur, with paintball munitions of different size, so that students could practice the sample collection using FNA [13], and small, soft plastic containers with pork-fat and bovine thymus, in which students could not only practice the FNA procedure, but also stain the retrieved cells and observe them using a microscope.

### 2.3. Student-Owned Animals

Students could bring their own companion animals to practical classes, voluntarily, without any age, sex, or breed requirements, if they were vaccinated and dewormed. Animals had to bear a leash to avoid eventual conflicts and muzzles were provided if needed. For ethical reasons, no painful or stressful manipulations were performed on students’ animals during classes. These practical classes are not considered as animal experimentation by current European and National Legislations (Law 113/2013). For the initial CEP (e.g., holding and restraining the animal), students first practiced with stuffed teddy dogs (Gosig Golden) and only utilized their own companion animals afterwards, assisted by the teacher. As previously mentioned, all possibly painful procedures were practiced only in simulation models.

### 2.4. Comparison between Model–Animal and Animal-Only Systems

Answers to the questionnaires and the exam pass rates in the final practical examination were compared. Regarding the latter, it was performed using Beagle dogs (AO) or with simulation models and their own animals (MA). An OSCE scoresheet was filled during the practical evaluation, and the comparison between groups focused on pass rates and final grades. Once students took their practical exam and received their grades, they answered a final questionnaire anonymously. This assessed the students’ own perception regarding the learning process, the importance of the knowledge acquired, and the degree of difficulty of the practical test.

Upon request, students were allowed to use the classroom facilities during out-of-school time. The number of requests per semester was compared in both systems.

### 2.5. Statistical Analysis

SPSS26 statistics software, version 26.0.0.0 (IBM, Armonk, NY, USA) was used for analysis. Normally distributed continuous data (final grades and number of requests to use the classroom out of school time) were analyzed using Student’s *t*-test, whereas other data were analyzed using the Mann–Whitney U test (questionnaire answers). A value of *p* < 0.05 was considered statistically significant.

## 3. Results

Most veterinary students had their own animals (62%) or had dogs from friends or family that could be taken to classes; therefore, it was easy to obtain one dog per group of two students during class. By adjusting the students’ class schedules, the animals came happily with their owners and stayed during classes without signs of stress. There were no conflicts between students’ animals in the MA system. Animals adapted fairly well to the class environment with other dogs and became more sociable over time. Furthermore, some students reported that these periods of socialization were beneficial for the animal’s behavior at home.

For most students, this was their first contextualized practical experience with different animals (young/old, thin/obese, calm/nervous) from various breeds. This contrasted to the practical classes of previous years (AO groups), which included only institutional Beagles, which were very similar to each other.

**Figure 3:**
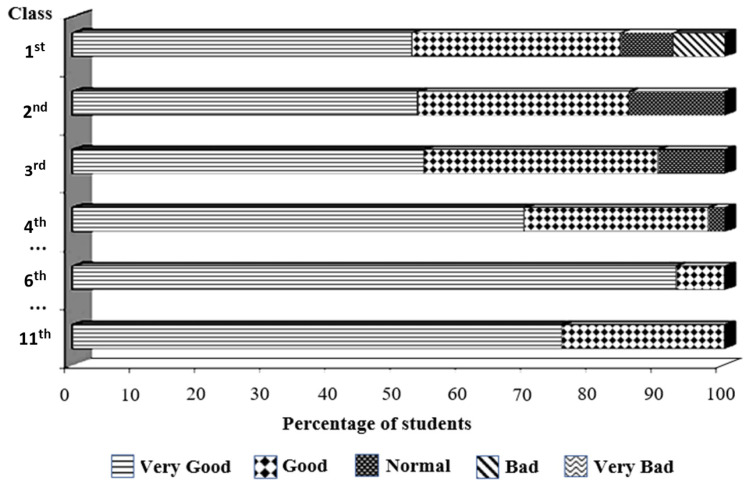
Students’ general evaluation of the classes using the mixed model system, as assessed using brief questionnaires in N3 (2020/21) and N4 (2021/22). In the first class, 8% of students rated the class as “Bad”, and another 8% rated it as “Normal”. By the fourth class, this percentage was reduced to 2%, and, thereafter, students appraised the classes as “Good” or “Very Good”.

The evaluation by students during the semester with the mixed system (MA) was positive, and their general impression improved over time, as assessed by using the brief questionnaires (Figure 3).

In the final questionnaire, when students appraised the amount of learning (1 to 5 Likert scale, 3 being the average), it was rated 4.71 ± 0.40 in the MA, which was superior (*p* < 0.001) to the AO classical system (4.31 ± 0.59) (Table 1). The interest was similar, being rated as 4.52 ± 0.49 in the MA and 4.47 ± 0.54 in the AO groups. Regarding the relevance of classes for their clinical activity as DVMs, as perceived by students, it was rated as 4.88 ± 0.21 in the MA group, similar to that of the AO system (4.79 ± 0.45). Regarding the final examination (difficulty, stringency, and fairness), no differences existed between the systems. The perception of students regarding the level of demand and difficulty of the exam were comparable for both groups of students. The pass rates obtained on the exam were similar: 81% to 88% in the AO system and 87% to 92% in the MA (Table 1). In this vein, the blended learning system was robust and maintained high standards of student learning, even under atypical conditions imposed by the recent COVID-19 pandemic.

**Figure 4:**
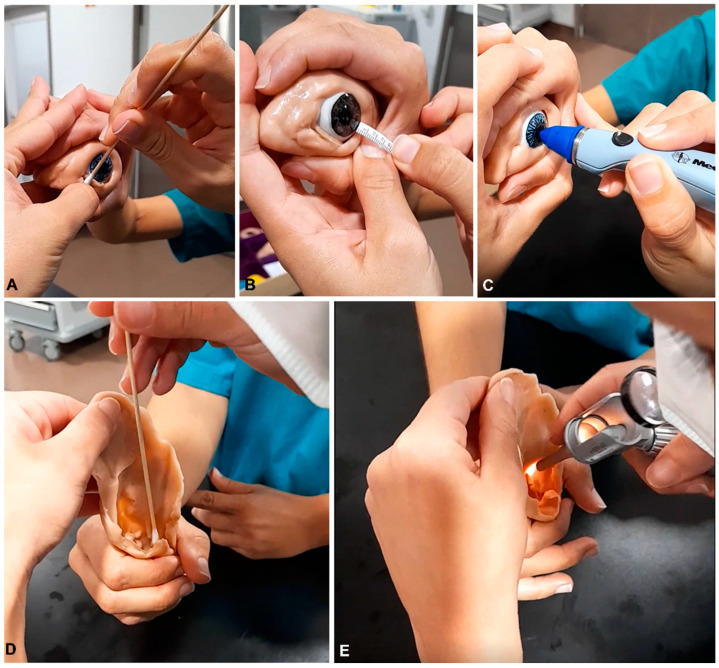
Students using the models to practice ophthalmology (**A**–**C**) and otoscopy procedures (**D**,**E**). By working in pairs, students obtained conjunctival swabs (**A**), performed the Shirmer’s test (**B**), and used the tonometer to assess the intraocular pressure (**C**). They also obtained ear canal swabs (**D**) and used the otoscope (**E**). In the ear canal, the tympanic membrane could be changed to represent simulated clinical conditions (inflammation and ruptured tympanum).

**Figure 5:**
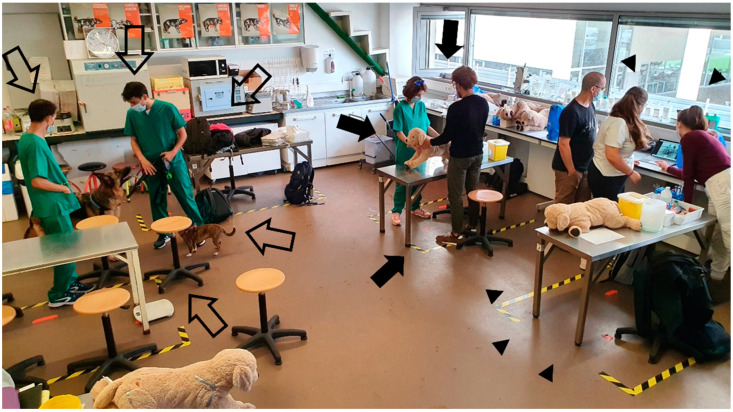
Students practicing out of school time, either using their own animals (block arrows) or stuffed teddy dogs (arrows and arrowheads). The group of students on the right (arrowheads) is also using video resources (supporting class material).

Regarding the simulation models, it was possible to create low-cost and accessible models with very high performances (Figure 4). All models could and were used by students in and out of school hours. The models (stuffed animals, soft silicone devices) were robust and suffered no apparent deterioration from repeated use by students during these two curricular years. The interest in the practical activities with the simulation models was comparable to that exhibited in the classical AO system. In some activities, such as the otoscopy and ophthalmology procedures, it was even greater, since students had higher chances of repeating more difficult procedures out of school hours, which was not possible with institutional animals in the AO system. During the two years, we had over 250 requests from students during the semester to use the classroom facilities out of school time in the MA system (compared with 80 requests in the previous years). In this facility, they could use the stuffed teddy dogs and silicone models and could bring their own animals, working in small groups (Figure 5). After the fourth week, students already recognized that they were using the models and animals regularly out of school time. More than half of the students had used more than one animal of different breeds to learn CEP (40% used two animals, 12% used three, and 2% had used more than three different animals). Despite having their own live animals available, a third of students used the models to practice out of school time. During the vacation periods, students also requested the models so that they could be used for learning at home. The number of requests was significantly higher in the MA system compared to AO.

## 4. Discussion

Currently, veterinary teaching stands in a crossroad between the classical perspective of using live animals and the more modern approach of using simulators and “Clinical Skills Labs” [6,8,14,15,16,17]. The use of live animals as “learning instruments” evokes ethical and financial dilemmas. Veterinary training itself seems to reverse the natural empathic and compassionate character of students and, probably, of later veterinarians. It has been described that veterinary students at the end of their 5 years of training tend to be less compassionate towards fear, boredom, hunger, and pain in animals [18], being less likely to treat animal pain than junior students [19]. Moreover, it has been shown that moral reasoning declines during the 5 years of veterinary education [20]. In the MA system, veterinary students tended to view the animals used in classes as their companion animals and not just as learning instruments imposed by teachers and administrators [20]. This reasoning probably accounts for the smooth transition from institutional animals to their own companion animals.

Using the students’ animals also has financial and animal welfare advantages. In our faculty, Beagles were used only for ≈26 weeks per year, meaning that the animals spend more time housed in kennels than in pedagogical activities. Regardless of the ethical issue that this entails, the pedagogical profitability of these animals is low, and the cost of food/personnel is very high. The cost of kenneling a dog varies substantially between countries, and marked differences may even exist within a country; still, it may cost between 4000 EUR (as in our institution, data not shown) to 6000 EUR per year [21]. Beyond the financial advantages of the MA system, there are animal welfare issues that must be considered. Spatial restriction is one of the factors that generates more stress [22,23,24], contributing to a decrease in animal welfare in kennels [24]. Numerous studies have already highlighted that dogs housed in kennel facilities for long periods of time experience suboptimal living conditions [22,25,26,27,28]. For instance, a higher incidence of chronic stress-related behavior problems has been described in dogs 4 to 8 weeks after admission to a kennel facility [22,28]. In this context, issues regarding the animal welfare of institutional animals can legitimately be raised, and there is a current “public opinion” advocating for a decrease in the use of animals among higher education institutions [7,11].

Shelter animals would be another alternative source, which has been assessed previously for teaching clinical-based subjects [10]. However, this would still require an established protocol with nearby shelters and would demand further logistics, such as the transport of students and a classroom facility at the shelter. Moreover, using shelter animals would still raise concerns regarding the repetition of painful or stressful procedures for learning purposes.

Many veterinary faculties in the world are reducing the number of animals used for teaching purposes, moving towards using simulation strategies and developing simulation devices [6,8,14]. Recently, the use of simulation-based methods in veterinary education has been reviewed, and many studies have shown that simulation prototypes reduce students’ stress when faced with real situations involving animals [29,30,31,32,33]. Repetitive practice is a major advantage of using simulators, and it has been shown that a repetitive practice in a model, prior to reproducing the task in a live animal, increases the self-confidence of students, develops their dexterity and motor skills, and reduces their levels of anxiety [33]. The use of complex simulators is not necessarily a prerequisite, since it has been shown in the surgical field that simple suture models can be as effective as more expensive devices [34,35,36,37]. Some high-tech simulation devices can be highly expensive, and some studies have been devoted to evaluating simpler and more economic solutions [16,17]. To the best of our knowledge, there are few experiences with low-cost and custom-based models in the context of veterinary semiology. Our simulation models cost from 7 EUR (Gosig Golden) to 90 EUR (more sophisticated eye and ear models) (Figure 1) and were endurable enough for students’ manipulation under intensive use (during classes and out of school time). The use of models beyond classes was also beneficial, since it has been shown that the learning of practical skills is enhanced if the tasks can be repeated in the following days [38]. This may account for the increased amount of learning reported by the students in the final questionnaire. Still, we are currently unable to disclose if such an effect was due to the use of models, their own companion animals, or both. If, on one hand, they practiced more on the models, then this probably helped to strengthen their self-confidence on the learned skills; on the other hand, the students became actively involved in the learning process by using their own companion animals, and this may have helped establishing a nurturing environment, in which students felt satisfied and may have learned more effectively. Furthermore, we may not exclude that the novelty effect may have led to an overvaluation of the students towards the practical skills they were learning. We cannot rule out the consequences of the Hawthorne effect, i.e., students behaving differently because they are being included in a study. The consequences of research participation for behaviors being investigated do exist, although little can be known for sure about the conditions under which they operate, their mechanisms, and their magnitudes [39]. Therefore, we will extend the present study to include more curricular years and will test this MA system in other institutions to have a wider global scope.

Our study has some limitations related to its single-center nature and learning outputs. Regarding the former, students were evaluated by a single instructor, not blinded to treatment/control status during the OSCEs, and this may be considered as a potential source of bias. Even if the eventual contribution towards the MA system cannot be excluded, this bias should be minimal considering that: (1) the instructor had a long experience in OSCE assessments, performing these evaluations for more than 20 years; (2) the students’ feedback assessed by questionnaires was comparable in both systems; (3) out-of-class deliberate practice by students increased significantly using the MA system. Deliberate practice sets the path to mastering clinical skills and probably increased students’ self-confidence in their practical skills, which will be needed later on in their fourth and fifth years of the DVM degree. It should be stressed that this study focused mostly on the first and second levels of the Kirkpatrick’s learning model, since we mostly analyzed the reaction and learning, dismissing the third and fourth levels, behavior and results, which are influenced by many external factors, such as economic and organizational contexts [5]. Even if it may be assumed that the MA system may have changed the students’ behavior, with students more compassionate towards fear, boredom, and pain in animals, we failed to assess this level. As for the fourth level, this could be addressed by evaluating the skills’ maintenance [40], which could have been achieved by repeating some of the OSCE evaluations in the following years or by evaluating their performance as veterinary practitioners later on [5].

## 5. Conclusion

In conclusion, we have shown that the MA system is as effective for learning and practicing CEPs in dogs as the classical AO model. Further studies are needed to elucidate the specific effects of the models included herein; however, the finding that the MA system is effective should be highlighted, as it may set the path towards a more “animal welfare”-driven veterinary education, which could improve the training and humane condition of veterinary students and, consequently, of future veterinary practitioners.

## Figures and Tables

**Table 1:** Comparison between students enrolled in the animal only system (AO), corresponding to 110 students from N1(2018/19) and N2(2019/20), and the students using the model–animal (MA) approach, comprising 121 students from N3(2020/21) and N4 (2021/22).

	Animal-Only (AO)	Model–Animal (MA)
Final questionnaire (Likert scale)		
Amount of learning	4.31 ± 0.59	4.71 ± 0.40 *
Interest	4.47 ± 0.54	4.52 ± 0.49
Relevance of classes	4.79 ± 0.45	4.88 ± 0.21
Pass rates at final exam	81–88%	87–92%

* *p* < 0.001.

## Data Availability

All data generated or analyzed during this study are included in this published article.
